# The impact on the bioenergetic status and oxidative-mediated tissue injury of a combined protocol of hypothermic and normothermic machine perfusion using an acellular haemoglobin-based oxygen carrier: The cold-to-warm machine perfusion of the liver

**DOI:** 10.1371/journal.pone.0224066

**Published:** 2019-10-23

**Authors:** Yuri L. Boteon, Richard W. Laing, Andrea Schlegel, Lorraine Wallace, Amanda Smith, Joseph Attard, Ricky H. Bhogal, Gary Reynolds, M. Thamara PR Perera, Paolo Muiesan, Darius F. Mirza, Hynek Mergental, Simon C. Afford

**Affiliations:** 1 Liver Unit, Queen Elizabeth Hospital, University Hospitals Birmingham NHS Foundation Trust, Birmingham, United Kingdom; 2 Centre for Liver and Gastrointestinal Research, Institute of Immunology and Immunotherapy, College of Medical and Dental Sciences, University of Birmingham, Birmingham, United Kingdom; 3 National Institute for Health Research (NIHR) Birmingham Biomedical Research Centre, University of Birmingham and University Hospitals Birmingham NHS Foundation Trust, Birmingham, United Kingdom; University of Cambridge, UNITED KINGDOM

## Abstract

**Introduction:**

The combination of hypothermic and normothermic machine perfusion (HMP+NMP) of the liver provides individual benefits of both techniques, improving the rescue of marginal organs. The aim of this study was to investigate the effect on the bioenergetic status and the oxidative-mediated tissue injury of an uninterrupted combined protocol of HMP+NMP using a single haemoglobin-based oxygen carrier (HBOC)-based perfusate.

**Methods:**

Ten discarded human donor livers had either 2 hours of dual hypothermic oxygenated perfusion (D-HOPE) with sequential controlled rewarming (COR) and then NMP using the HBOC-based perfusate uninterruptedly (cold-to-warm group); or 2 hours of hypothermic oxygenated perfusion (HOPE) with an oxygen carrier-free perfusate, followed by perfusate exchange and then NMP with an HBOC-based perfusate. Markers of liver function, tissue adenosine triphosphate (ATP) levels and tissue injury were systematically assessed.

**Results:**

The hypothermic phase downregulated mitochondrial respiration and increased ATP levels in both groups. The cold-to-warm group presented higher arterial vascular resistance during rewarming/NMP (*p* = 0.03) with a trend of lower arterial flow (*p* = 0.09). At the end of NMP tissue expression of markers of reactive oxygen species production, oxidative injury and inflammation were comparable between the groups.

**Conclusion:**

The uninterrupted combined protocol of HMP+NMP using an HBOC-based perfusate—cold-to-warm MP—mitigated the oxidative-mediated tissue injury and enhanced hepatic energy stores, similarly to an interrupted combined protocol; however, it simplified the logistics of this combination and may favour its clinical applicability.

## Background

*Ex situ* machine perfusion (MP) demonstrates superior preservation for extended criteria donor (ECD) livers [1–3]. The MP field has largely focused on oxygenated hypothermic MP (HMP) and normothermic MP (NMP) to date, and both MP techniques have reported benefits over standard static cold storage [3–5]. There remains controversy as to whether markers of liver injury and function are comparable between HMP and NMP [6, 7]. Preclinical and clinical studies have recently suggested that a combined protocol of HMP and NMP is feasible and it may derive individual benefits of both techniques [8–11].

The inability to utilise a red blood cell-based perfusate during HMP, owing to the risk of sludging [12] and haemolysis [9] at hypothermia as well as the need for an oxygen carrier during NMP, imposes the need for perfusate exchange during the perfusion process if one wishes to combine both techniques, as it was previously done by our research group [8]. This interruption in the perfusion process adds an unnecessary ischaemic time to the organs, which may hinder the implementation of the combined protocol of HMP and NMP.

Haemoglobin-based oxygen carriers (HBOC) have an advantage that can potentially be used under a wide range of temperatures with reports on HMP, subnormothermic MP at 20 °C and NMP [9, 13, 14]. In addition, it would allow gradual rewarming of the organs (controlled oxygenated rewarming [COR]), which has also been suggested to mitigate reperfusion injury and improve graft function post-transplantation [11, 15, 16].

It was then hypothesised that the use of a single HBOC-based perfusate for a combined perfusion from cold to warm might eliminate entirely any concerns of exposing the organs to additional unnecessary ischaemia time, required for perfusate exchange, optimising the logistics of a combined perfusion, thereby increasing its clinical applicability. This hypothesis was tested clinically in two recent studies [9, 11]. While the authors have shown the clinical feasibility of this combined protocol, they were not able to investigate markers of oxidative tissue injury, activation of the inflammatory cascade and replenishment of hepatic adenosine triphosphate (ATP) levels. These analyses are crucial because the hypothermic phase in the combined protocol aims specifically to increase hepatic ATP content and mitigate the harmful impact of the oxidative tissue injury [3].

The aim of this study was to investigate the effect on oxidative tissue damage, activation of inflammatory markers and hepatic ATP content of a combined protocol of HMP and NMP—dual hypothermic oxygenated perfusion (D-HOPE) with sequential slow COR and NMP—using a single HBOC-based perfusate throughout; and compare these results with those from a previously published interrupted combined protocol which has used an HBOC-based perfusate only for the NMP phase, requiring perfusate exchange during the perfusion run [8].

## Materials and methods

**Fig 1:**
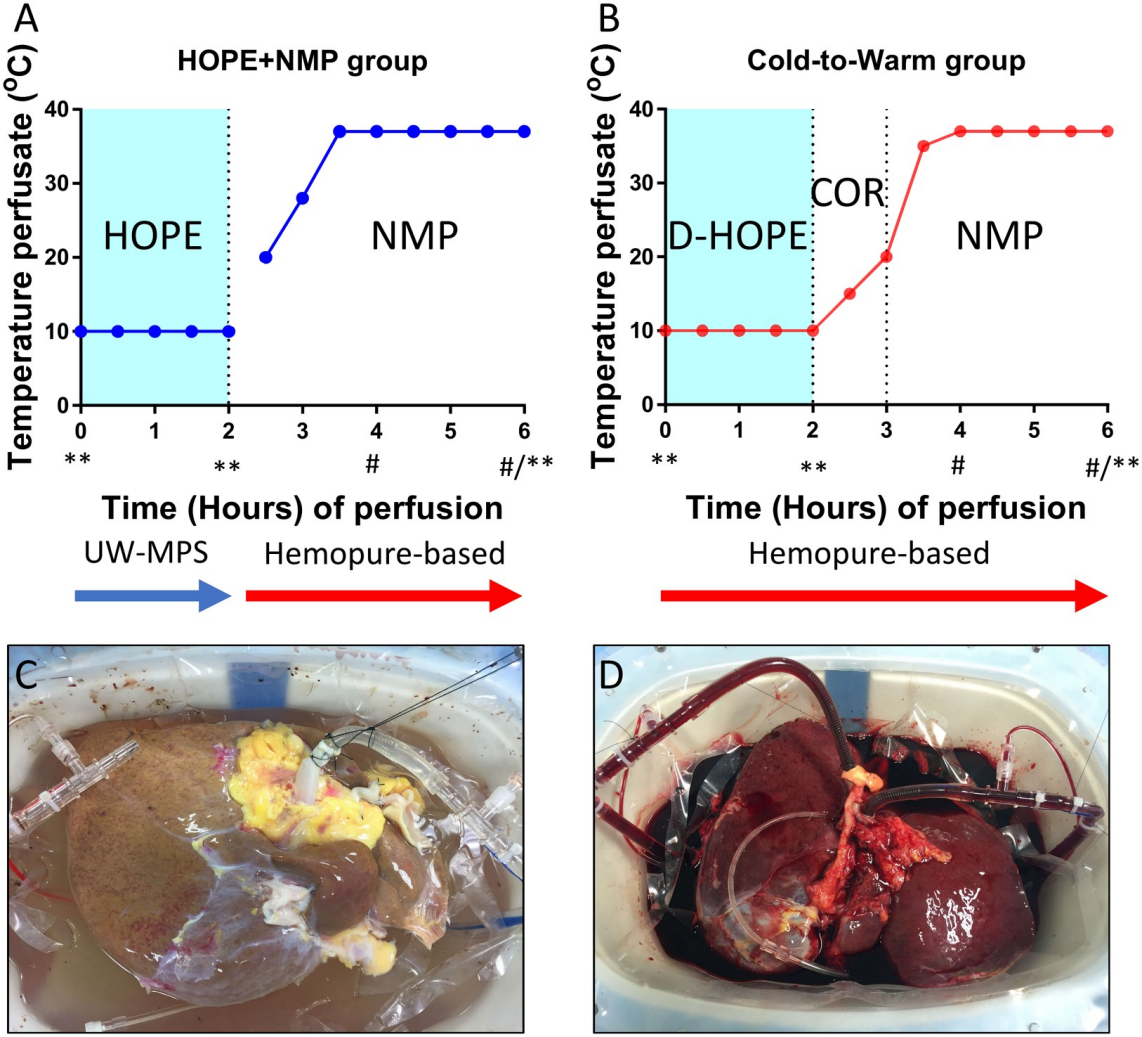
Study design. Donor human livers had standard procurement, they were cold flushed and stored. Once rejected for transplantation, they were offered for research and consecutively allocated to the two experimental groups. Image A shows the protocol for the HOPE+NMP group, livers had hypothermic oxygenated perfusion (HOPE) using Belzer MPS^®^ UW Machine Perfusion Solution for 2 hours. HOPE was performed at 10 °C via the portal vein only, as represented on image C. After 2 hours, the perfusate was changed to an acellular haemoglobin-based oxygen carrier (HBOC) Hemopure^®^ (HBOC-201, Hemoglobin^®^ Oxygen Therapeutics LLC, Cambridge, USA)-based perfusate for the rewarming and normothermic machine perfusion (NMP). The livers from the cold-to-warm group (Image B) were fully cannulated at the start of the perfusion, including portal vein, hepatic artery and common bile duct (Image D). They received the HBOC-based perfusate from time 0. For this group, the livers had 2 hours of dual hypothermic oxygenated perfusion (D-HOPE) at 10 °C followed by 1 hour of slow rewarming to 20 °C (controlled oxygenated rewarming [COR]) and then NMP. Menghini and wedge biopsies were collected at time 0, 2 and 6 hours (**) and immediately fixed in formalin or snap-frozen in liquid nitrogen. Blood gas analysis was carried out and perfusate was sampled at 30 min time intervals throughout. In addition, bile production was measured at time 4 and 6 hours (#) and analysed at 6 hours.

Ten discarded human livers were sequentially allocated to two experimentally matched groups: (1) the HOPE+NMP group that had two hours of hypothermic oxygenated perfusion (HOPE), using a Belzer MPS^®^ UW Machine Perfusion Solution (UW-MPS) (Bridge to Life, London, UK) as the perfusate, followed by NMP employing an HBOC-based perfusate (Hemopure^®^—haemoglobin glutamer-250 [bovine]; HBOC-201, Hemoglobin^®^ Oxygen Therapeutics LLC, Cambridge, USA) (previously published group [8]); and (2) the cold-to-warm group—this group had an HBOC-based perfusate starting at time 0 that permitted simplifying changes to the protocol: hepatic artery (HA) and portal vein (PV) were cannulated during commencement and D-HOPE was carried out for the hypothermic phase. Following this, the organs were gradually rewarmed (COR) and then had NMP for viability assessment. The total perfusion time was six hours for both groups. Perfusate, bile and tissue biopsies were sampled systematically. The detailed sampling protocol is presented in Fig 1.

### Source of discarded human livers

All livers were retrieved with the intention of clinical transplantation according to the National Organ Retrieval Service standards. After the procurement, they were stored on ice in Belzer University of Wisconsin (UW^®^) Cold Storage Solution (Bridge to Life) and then rejected for transplantation by all transplant centres in the United Kingdom and offered for research by the National Health Service Blood and Transplant (NHSBT) coordinating office. Specialist nurses in organ donation obtained consent to use donor tissue for research as part of the consent process for standard clinical organ donation. None of the donor organs were from a vulnerable population and all next of kin provided written informed consent that was freely given. The study’s ethical approval was obtained from the London-Surrey Borders National Research Ethics Service and the NHSBT Ethics Committee (references 13/LO/1928 and 06/Q702/61, respectively).

### Organ preparation and machine perfusion procedure

After arrival at our centre, the livers had a standard bench preparation as described elsewhere [17] followed by flushing with two litres of 5% glucose solution. The Liver Assist Device (Organ Assist, Groningen, Netherlands) was applied for MP. The perfusate temperature and perfusion pressures were set by the operator as specified in the Supplementary Material. The measured flow rates and calculated resistances, as shown on the device’s display in real time, were recorded every 30 minutes. Oxygen was supplied via a Sechrist air/oxygen blender (S3500CP‐G, Inspiration Healthcare, Ltd., Leicester, United Kingdom). The fraction of inspired oxygen was set initially at 30% with a flow of 1–2 L/min and adjusted to achieve the target oxygen perfusate pressure for each modality of perfusion as specified in the Supplementary Material. A detailed description of the techniques underlying MP performed in this study is presented in the Supplementary Material, and the composition of the HBOC-based perfusate is found in S1 Table.

### Assessment of liver metabolism

The perfusate was sampled every 30 minutes throughout the perfusion in both groups and analysed immediately for blood gases or snap‐frozen and stored for later analyses. Bile produced during the perfusion was collected via a 12‐Fr silicon tube inserted into the common bile duct, weighed at 4 and 6 hours of MP and immediately analysed at 6 hours. Both, bile and perfusate samples, were assessed using a Cobas b 221 (Roche Diagnostics, Indianapolis, IN) point‐of‐care system blood gas analyser. Perfusate parameters included partial pressure of oxygen (PO_2_) and partial pressure of carbon dioxide (PCO_2_), pH, base excess, bicarbonate, oxygen saturations, haemoglobin, haematocrit, glucose and lactate concentrations. For bile samples, bile pH and glucose concentrations were analysed.

Oxygen uptake was calculated during HOPE perfusion (oxygen carrier-free-based perfusate) as the oxygen inflow minus the outflow in kPa, corrected by liver weight. For the HBOC-based perfusate, the oxygen consumption was calculated from the difference between the oxygen content after the oxygenator and return into the oxygenator within the venous circuit. Oxygen content was calculated as the sum of the free dissolved oxygen fraction and the haemoglobin-bound oxygen fraction (equation located in the Supplementary Methods) as described elsewhere [18].

The recovery of appropriate liver metabolism was assessed during NMP by our unit’s published criteria. The major criterion was perfusate lactate levels < 2.5 mmol/L, in association with the evidence of bile production, glucose consumption, stable vascular flows and homogeneous parenchymal perfusion [19, 20].

### Histopathological assessment of hepatocyte injury

Liver biopsies obtained prior to and at the end of perfusion were immediately fixed in formalin, processed and embedded in paraffin. Thereafter, 4 μm sections were cut and stained with haematoxylin and eosin (H&E) as well as periodic acid Schiff (PAS). H&E sections were semiquantitatively graded for ischaemic-type coagulative necrosis, macrovesicular steatosis (large lipid droplets filling up the hepatocytes and displacing the nucleus to the periphery) and pre-existing liver disease. The PAS-stained sections were scored for the percentage of positive areas of glycogen, and the variation between the beginning and end of the perfusion was compared across groups. Histological assessment was blinded and conducted by an experienced liver transplant pathologist.

### Immunohistochemical assessment of oxidative stress and tissue inflammation

Immunohistochemistry was performed on formalin-fixed paraffin-embedded sections to assess surrogate markers of oxidative injury and tissue inflammation. Uncoupling protein 2 (UCP2) was assessed for reactive oxygen species (ROS) production [21–23]. Oxidative tissue injury was evaluated by the expression of 4-hydroxynonenal (4-HNE), a product of lipid peroxidation in cells. For the assessment of tissue inflammation, we analysed the following markers: (1) cluster of differentiation (CD)14, a lipopolysaccharide receptor which is part of the toll-like receptor 4 signalosome [24–26]; (2) CD11b is an integrin on the surface of leukocytes; upregulation of its expression indicates activation of the cells by substances, including ROS [27, 28]; and (3) the vascular cell adhesion molecule 1 (VCAM-1), a cell adhesion molecule expressed in cytokine-activated endothelial cells that mediates leukocyte transendothelial migration. All primary antibodies were detected using specific ImmPRESS^™^ Excel Amplified HRP Polymer Staining kits (Vector Laboratories, Burlingame, CA, USA) specific to the respective mouse or rabbit immunoglobulin isotype. A list of primary antibodies and dilutions used is provided in the Supplementary Material.

Four images of each section, excluding the edges, were randomly selected for analysis (×400 magnification). A semiquantitative scoring system, the modified immunoreactive score (IRS) [29], was obtained by multiplying the score for intensity (0: no colour reaction; 1: mild reaction; 2: moderate reaction; 3: intense reaction) and distribution (0: no positive cells; 1: <10% positive cells; 2: 10–50% positive cells; 3: 51–80% positive cells; 4: >80% positive cells) to obtain a final score between 0 and 12. The change in overall tissue expression of staining (ΔIRS) was determined by subtracting the IRS score after 6 hours of perfusion from the score prior to perfusion: negative values indicated a decrease and positive values an increase in the expression of staining.

### Assessment of tissue adenosine triphosphate (ATP) levels

Quantification of ATP was carried out by homogenisation of liver tissue. The concentration was determined with the ATP Bioluminescent Assay kit (FLAA, Sigma-Aldrich Inc, St Louis, USA). More details are provided in the Supplementary Material.

### Statistical analysis

Continuous variables were expressed as median with interquartile range (IQR) and categorical variables as the absolute number with percentage frequencies. Comparisons between groups were performed via Fisher’s exact test for categorical variables, the Mann—Whitney *U* test for independent continuous variables and the Wilcoxon signed-rank test for repeated measurements over time on the same sample. The statistical level of significance was set at *p* < 0.05. GraphPad Prism (version 6.04 for Windows, GraphPad Software, La Jolla, USA) software was employed for graph creation and all statistical analyses were carried out with the Statistical Package for the Social Sciences version 22 software (IBM Corp, Armonk, NY).

## Results

### Donor demographics and organ characteristics

The median donor age for all livers was 54 years (IQR: 51–55), donor body mass index 31 kg/m^2^ (24–36) and donor risk index 2.1 (2.0–2.6). Median cold ischaemia time was 663 minutes (510–712) and warm ischaemia time for donors after circulatory death (DCD) 8 minutes (7–31). Detailed donor demographics and donor organ characteristics are presented in Table 1. Each experimental group had three DCD livers and the groups were comparable regarding donor and liver characteristics (Table 2).

**Table 1:** Donor demographics, liver characteristics and machine perfusion parameters.

Liver number	Cold-to-warm 1	Cold-to-warm 2	Cold-to-warm 3	Cold-to-warm 4	Cold-to-warm 5	HOPE+NMP 1	HOPE+NMP 2	HOPE+NMP 3	HOPE+NMP 4	HOPE+NMP 5
**Donor information**										
Age	73	51	55	57	52	54	50	54	38	55
Donor type	DBD	DCD	DBD	DCD	DCD	DCD	DBD	DBD	DCD	DCD
Sex	Male	Male	Male	Female	Female	Male	Female	Female	Male	Female
Height (cm)	164	189	172	160	160	179	158	170	183	160
Bodyweight (kg)	90	76	85	70	90	123	60	87	85	90
Body mass index (kg/m^2^)	33	21	29	27	35	38	24	30	25	35
US—Donor risk index	2.0	2.1	2.0	2.5	2.9	2.5	2.0	1.9	1.8	3.0
UK—donor liver index	1.4	1.8	1.1	1.9	2.1	1.8	1.1	1.0	1.8	1.9
ET—donor risk index	2.0	2.7	2.0	2.5	2.8	2.9	2.2	1.9	2.3	2.8
Peak ALT (IU/L)	33	74	72	135	81	189	14	271	37	741
Days on ventilator	4	3	2	11	4	5	2	2	4	4
Co-morbidities and risk history	HTN	Smoker	Diabetes (type 2)	Alcohol misuse	Smoker, HTN	Diabetes (type 2) HTN	Smoker, Alcohol misuse	Diabetes (type 1), Smoker	Active Smoker	HTN
Cause of death	HBD	HBD	ICH	ICH	HBD	HBD	ICH	ICH	ICH	HBD
**Liver characteristics**										
Liver weight (g)	1652	1900	1920	2140	1598	2600	1838	2060	1753	1935
Donor warm ischaemic time (minutes)	NA	8	NA	10	14	31	NA	NA	42	8
Cold ischaemic time (minutes)	532	645	793	430	934	497	682	491	660	510
Retrieval location	Regional	Extra-zonal	Extra-zonal	Regional	Extra-zonal	Extra-zonal	Extra-zonal	Extra-zonal	Extra-zonal	Extra-zonal
Reason for clinical rejection	Donor cancer	Steatosis	Steatosis	Steatosis	Donor cancer	Poor flush	Steatosis	Steatosis	Poor flush	Steatosis, poor flush
Large droplets macrovesicular steatosis (paraffin sections)	0%	0%	20%	40%	0%	10%	0%	15%	3%	30%
**Machine perfusion parameters—throughout entire duration of perfusion**										
Lactate (mmol/L)										
Highest	6.7	9.1	12.0	12.6	10.2	9.1	8.9	10.4	4.6	7.8
Lowest	0.3	0.7	5.1	2.4	0.2	0.6	1.1	1.8	0.2	0.8
At the end of 6-hour perfusion	0.3	0.7	6.3	2.4	0.2	0.6	1.1	1.8	0.2	0.8
Total Bile production (g)	5.0	3.2	0.0	10.0	0.0	57.0	0.0	0.0	15.9	24
Mean Arterial flow (mL/min)	92	102	132	77	127	292	299	234	152	398
Mean Portal vein flow (mL/min)	636	619	390	322	451	1412	1523	1602	1406	1582
Mean liver mass perfusion (mL/g/min)	0.4	0.4	0.3	0.2	0.4	0.7	1.0	0.9	0.9	1.0
Viability criteria met	Yes	Yes	No	Yes	Yes	Yes	Yes	Yes	Yes	Yes

**Abbreviations**: HOPE: hypothermic oxygenated perfusion of the liver; NMP: Normothermic machine perfusion of the liver; DCD: donation after circulatory death; DBD: donation after brain death; US: United States; UK: United Kingdom; ET: Eurotransplant; ALT: alanine aminotransferase; HTN: hypertension; ICH: intracranial haemorrhage; HBD: hypoxic brain damage; NA: not applicable.

**Note**: Donor warm ischaemic time was defined as the interval between the systolic blood pressure less than 50 mmHg or/and arterial oxygen saturation to less than 70% to commencing the aortic cold perfusion in the donor.

**Table 2:** Donor demographics, liver characteristics and machine perfusion data.

Liver number	Cold-to-Warm (*n* = 5)	HOPE+NMP (*n* = 5)	*p*-value
**Donor information**			
Age, years	55 (52–57)	54 (46–54)	0.87
DCD livers	3 (60%)	3 (60%)	>0.99
Sex, male	3 (60%)	2 (40%)	0.53
Height (cm)	164 (160–172)	179 (169–181)	0.11
Bodyweight (kg)	85 (76–90)	90 (87–106)	0.98
Body mass index (kg/m^2^)	29 (27–33)	35 (30–37)	0.73
Donor risk index	2.1 (2.0–2.5)	2.5 (2.2–2.7)	0.20
UK donor liver index	1.8 (1.2–2.0)	1.8 (1.0–1.8)	0.55
ET donor risk index	2.5 (2.0–2.8)	2.3 (2.0–2.8)	0.84
Peak ALT (IU/L)	74 (72–81)	189 (37–271)	0.18
Days on ventilator	4 (3–4)	4 (2–4)	0.45
**Liver characteristics**			
Liver weight (g)	1900 (1749–2020)	1935 (1838–2060)	0.58
Donor warm ischaemic time (minutes)	8 (6–11)	31 (19–36)	0.10
Cold ischaemic time (minutes), DCD	705 (537–789)	682 (586–708)	0.86
Cold ischaemic time (minutes), DBD	658 (523–793)	586 (490–682)	0.93
Macrovesicular steatosis (%)	0 (0–30)	10 (1–22)	0.97
**Machine perfusion parameters—throughout entire duration of perfusion**			
Lactate (mmol/L)			
Highest	10.2 (9.1–12.0)	8.9 (7.8–9.1)	0.37
Lowest	0.7 (0.3–2.4)	0.8 (0.6–1.1)	0.45
Last	0.7 (0.3–2.4)	0.8 (0.6–1.1)	0.45
Total Bile production (g)	4 (0–6)	16 (0–40)	0.20

**Abbreviations**: NMP: normothermic machine perfusion of the liver; HOPE: hypothermic oxygenated perfusion of the liver; DCD: donation after circulatory death; UK: United Kingdom; ET: Eurotransplant; ALT: alanine aminotransferase; DBD: donation after brain death.

**Note**: Continuous variables presented as median and interquartile range, dichotomous variables as absolute number and percentage. Donor warm ischaemic time was defined as the interval between the systolic blood pressure less than 50 mmHg or/and arterial oxygen saturation to less than 70% to commencing the aortic cold perfusion in the donor.

### Stability of an HBOC-based perfusate at variable temperatures

**Fig 2:**
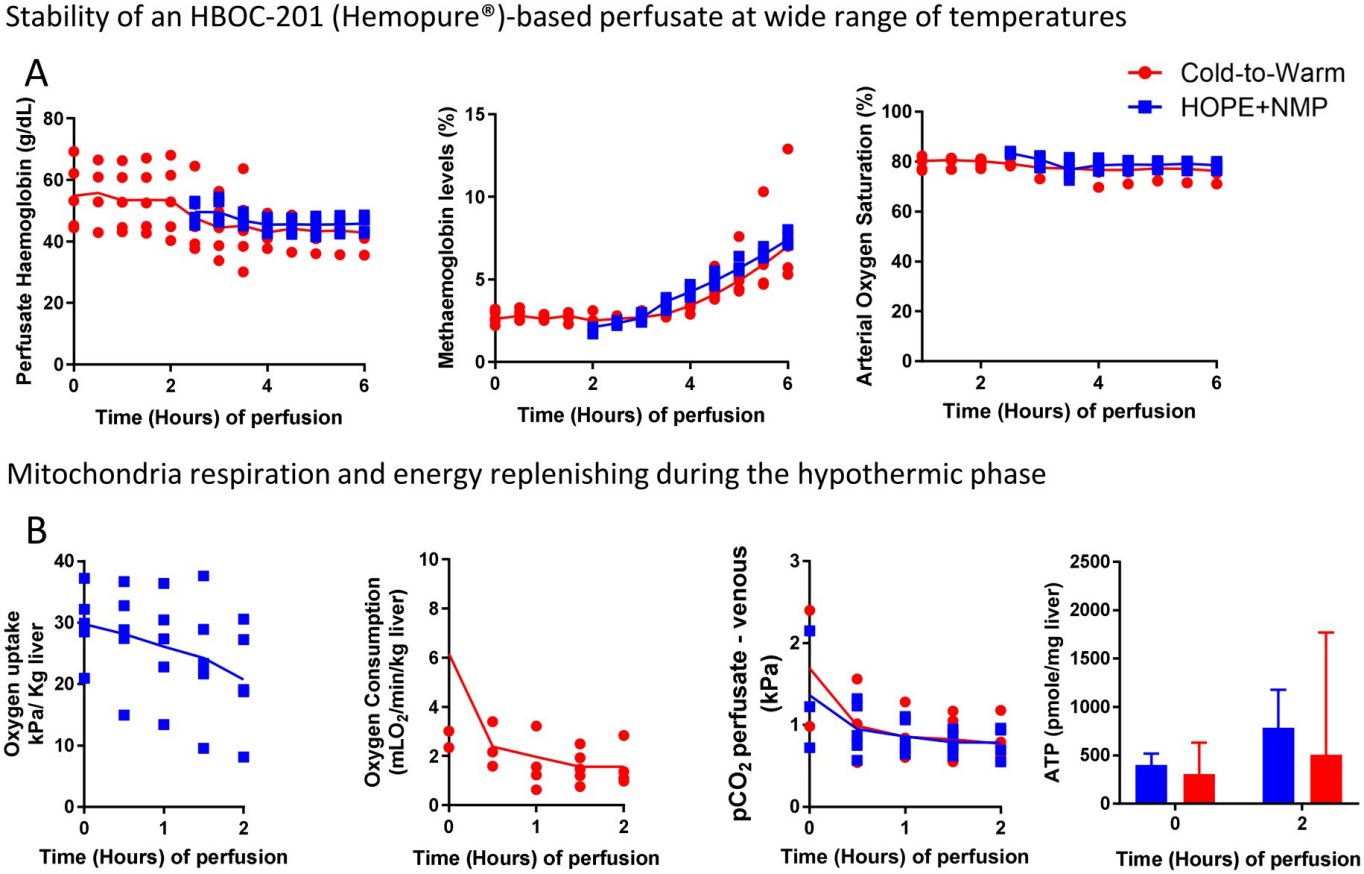
Stability of HBOC-based perfusate at different temperatures and its impact on mitochondrial function at hypothermic temperatures. Panel A shows on the left-hand graph that haemoglobin levels were stable during the D-HOPE phase, and after rewarming, they were similar to the HOPE+NMP group that received fresh HBOC-based perfusate. Methaemoglobin levels (middle graph) were stable during D-HOPE and increased slowly during the rewarming, reaching comparable levels to the HOPE+NMP group during the normothermic phase. Despite the increase in the levels of methaemoglobin, arterial oxygen saturation was constant throughout the perfusion. Panel B compares markers of mitochondrial respiration between the study groups. Both groups presented a downward trend in oxygen requirements (oxygen uptake—HOPE+NMP group and oxygen consumption—cold-to-warm group), with a similar drop in the release of carbon dioxide (CO_2_) in the perfusate. Reflecting an efficient mitochondrial oxidative function, both groups replenished adenosine trisphosphate (ATP) stores during the hypothermic phase. In the graphs, dots represent individual organs at the time points, and the line is the median of the values for each group. For the bar graph, the median and interquartile range are represented.

Haemoglobin levels of the HBOC-perfusate were comparable between the start and end of the hypothermic phase (D-HOPE) (53.2 [44.7–65.6] vs. 52.8 [42.5–64.7] g/dL, *p* = 0.59). At the beginning of the COR phase, antibiotics and other supplements (specified in S1 Table) were added to the HBOC-based perfusate, to supply the increasing metabolic demand of the organs over the next perfusion stages, and a dilutional effect was observed between 2 and 3 hours. The median haemoglobin levels were similar at the end of NMP between the groups (HOPE+NMP 46.7 [42.9–47.7] vs. cold-to-warm 47.5 [38.2–50.4] g/dL). Methaemoglobin (MetHb) levels were stable during the D-HOPE and COR phases and then rose with the rewarming/NMP phase. Despite this trend, the levels of oxygen saturation were constant. Details are found in Fig 2.

### The effectiveness of an HBOC-based perfusate on mitochondrial respiration and energy replenishing during the hypothermic phase

During the HOPE phase, the livers exhibited downregulation of mitochondrial respiration with a decrease in the oxygen uptake rate and CO_2_ release with the perfusate. Similarly, during D-HOPE, the livers experienced a fall in oxygen consumption rate from time 0 to 2 hours (2.68 [2.35–3.02] vs. 1.24 [1.11–4.47] mLO_2_/min/kg liver, *p* = 0.10) with a drop in pCO_2_ levels, reaching similar levels at 2 hours (HOPE+NMP 0.8 [0.6–1.0] vs. cold-to-warm 1.2 [1.0–1.7] kPa, *p* = 0.28). Pre-perfusion ATP levels were comparable between groups (*p* > 0.99). At the end of 2 hours HMP, ATP levels rose 1.8-fold in the HOPE+NMP group and 2.5-fold in the cold-to-warm group. See further details in Fig 2.

### The effect of hypothermic perfusion on the rewarming and normothermic phases

**Fig 3:**
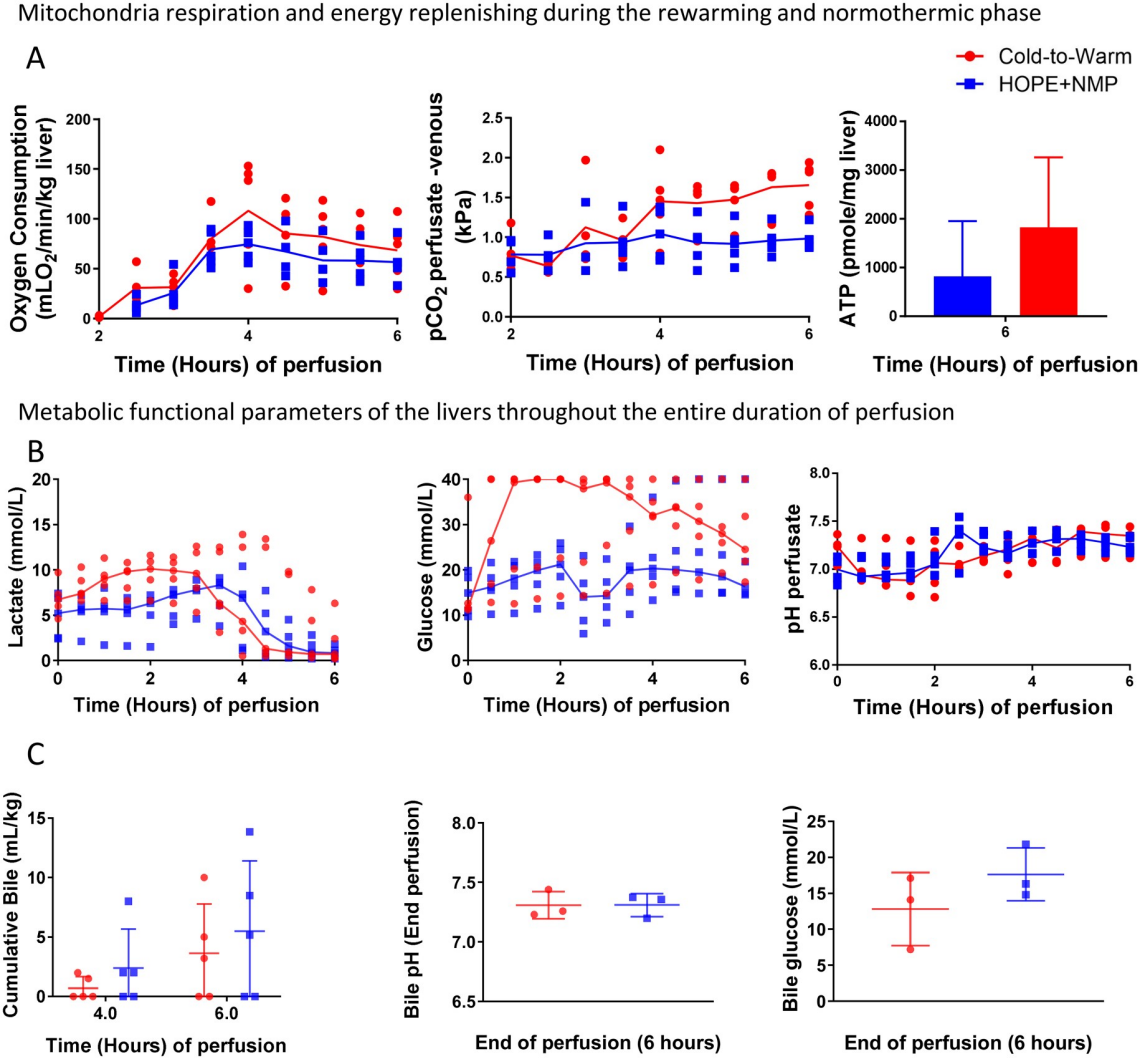
Mitochondrial and hepatic functional markers during the normothermic phase. Panel A shows a similar incremental rate of oxygen consumption during the rewarming period, which finally culminated in a higher peak at the beginning of the normothermic phase for the cold-to-warm group. The rate of carbon dioxide (CO_2_) release in the perfusate followed a similar trend to the oxygen consumption, and the adenosine triphosphate (ATP) levels reached higher figures at the end of the 6 hours of perfusion in the cold-to-warm group than with the HOPE+NMP. Panel B represents parameters of metabolic function of the organs during the perfusion. During the hypothermic phase, lactate levels increased slightly for the livers that had D-HOPE and were constant throughout HOPE. After rewarming, the lactate peak was comparable between groups, and then the lactate clearance was more effective in the cold-to-warm group resulting in similar levels at the end of the perfusion. There was evidence of glycogenolysis at the beginning of the D-HOPE perfusion, and thereafter the organs start to consume glucose during the NMP phase. For the HOPE+NMP group, there was a sudden drop related to perfusate change at 2-hour perfusion and then the livers start to metabolise glucose during the NMP phase. Perfusate pH was similar between groups during the perfusion. Three livers in each group produced bile during the perfusion (Panel C). The bile pH was comparable between them, as was the glucose content. In the graphs, dots represent individual organs at the time points, and the line is the median of the values for each group. For the bar graph, the median and interquartile range are represented.

During the COR phase, oxygen consumption increased slightly and reached 4.06 mLO_2_/min/kg liver (3.66–4.47). After rewarming during the NMP phase, despite not being statistically significant, the peak of oxygen consumption was higher for the cold-to-warm group (138.60 [52.26–149.19] vs. 75.89 [59.22–89.72] mLO_2_/min/kg, *p* = 0.22). The CO_2_ levels increased steadily for the cold-to-warm group during the NMP phase and were stable for the HOPE+NMP group. At the end of the perfusion run, the levels of ATP increased 2.7-fold in the HOPE+NMP group and 5.8-fold in the cold-to-warm group (Fig 3).

### Vascular flow dynamics

Median arterial vascular resistances were high during D-HOPE (0.3 mmHg/mL/min/kg) and decreased slightly during the COR phase (0.2 mmHg/mL/min/kg). When the arterial perfusion was started in the HOPE+NMP group, HA vascular resistance was lower than in the cold-to-warm group (0.2 [0.1–0.3] vs. 0.4 [0.3–0.7] mmHg/mL/min/kg, *p* = 0.03). It remained significantly lower in the HOPE+NMP group until 5 hours of perfusion and then decreased for the cold-to-warm group, reaching a non-significant difference at the end of the normothermic phase (0.1 [0.1–0.2] vs. 0.2 [0.1–0.2] mmHg/mL/min/kg, *p* = 0.22). Despite a non-significant difference at the beginning, at the end of the NMP, the median flow rates for HA were comparable between groups (HOPE+NMP 365 [256–388] vs. cold-to-warm 310 [250–332] mL/min).

**Fig 4:**
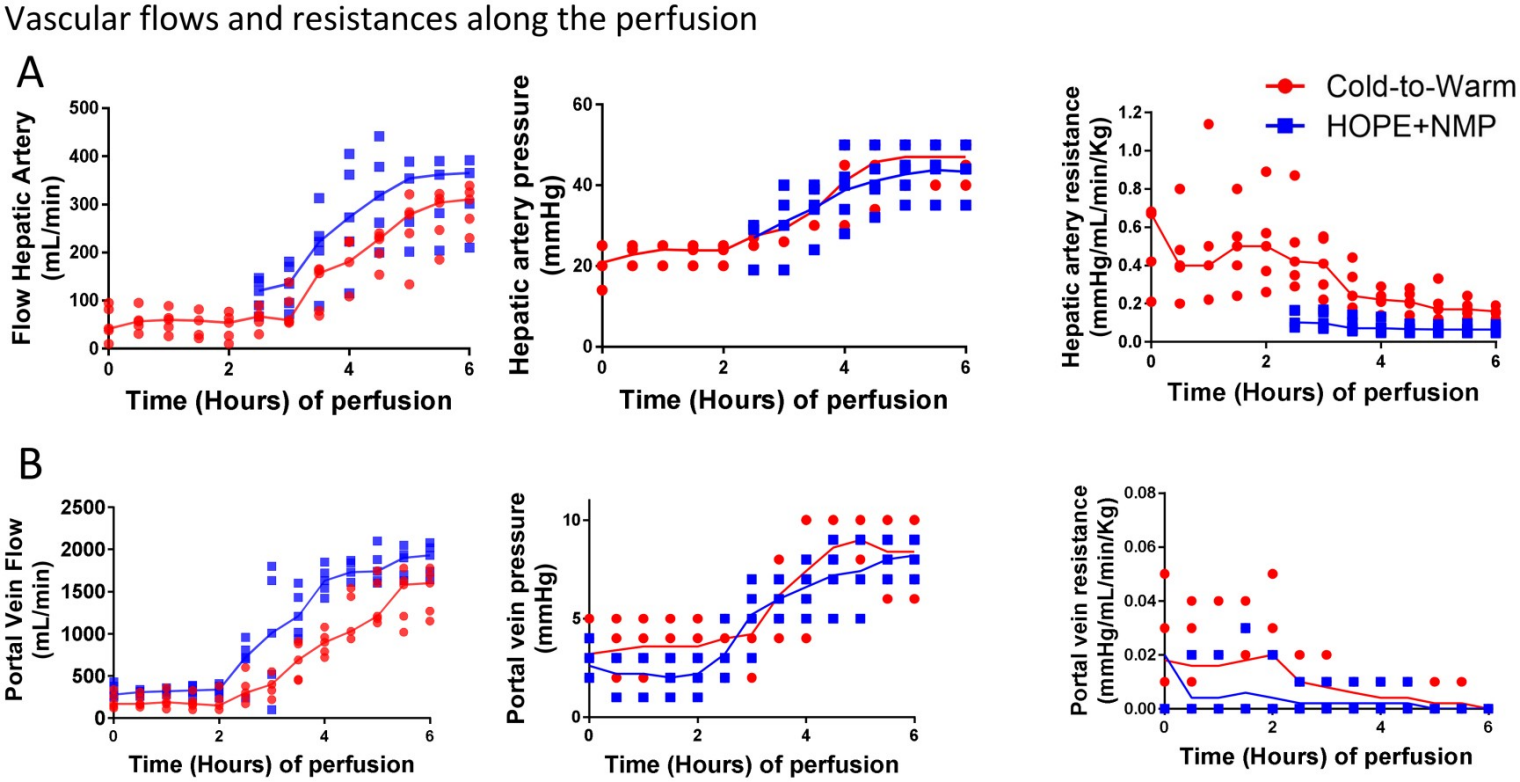
Vascular parameters throughout the perfusion protocols. Panel A shows hepatic artery vascular parameters. The flow was higher for the HOPE+NMP group, despite slightly higher pressures used for the cold-to-warm group in an attempt to overcome the higher vascular resistance. A similar trend was seen for the portal vein vascular parameters (Panel B). The cold-to-warm group had higher vascular resistance mainly during the hypothermic phase, which improved during the rewarming. The higher vascular resistance required increases in the perfusion pressures, although the vascular flows were still lower during the initial period of the NMP phase. In the graphs, dots represent individual organs at the time points and the line is the median of the values for each group.

A maximum portal venous pressure of 5 mmHg was used for the cold-to-warm group to overcome the higher vascular resistance (Fig 4). This was kept at 3 mmHg for the HOPE+NMP group. The difference in flow rates for PV did not reach statistical significance; however, values were higher for the HOPE+NMP group at the beginning of the HMP phase (280 [175–405] vs. 170 [130–300] mL/min) and at the end (320 [185–360] vs. 150 [125–305] mL/min). At the end of the NMP, both groups had adequate PV flow (HOPE+NMP 1740 [1140–1675] vs. cold-to-warm 1600 [1210–1740] mL/min). Fig 4 portrays the vascular flow and resistance over the time of the perfusion.

### Assessment of metabolic parameters during normothermic machine perfusion

Perfusate lactate levels were similar at the start of the perfusion (HOPE+NMP 4.7 [2.5–7.4] vs. cold-to-warm 6.6 [4.9–9.1] mmol/L, *p* = 0.31). Those levels were constant for the HOPE+NMP group and increased for the cold-to-warm group, reaching higher levels at the end of the hypothermic phase (5.5 [2.4–6.9] vs. 9.8 [7.5–11.3] mmol/L, *p* = 0.01). After rewarming, the lactate peak was similar between groups (HOPE+NMP 9.1 [5.7–10.0] vs. cold-to-warm 10.2 [7.9–12.3] mmol/L, *p* = 0.55). Both groups metabolised lactate effectively with rewarming and NMP, achieving comparable values at the end of the perfusion (HOPE+NMP 0.8 [0.3–1.4] vs. cold-to-warm 0.7 [0.2–1.4] mmol/L, *p* = 0.84).

Venous perfusate glucose was also comparable between groups at the commencement of the hypothermic phase (HOPE+NMP 10.3 [9.6–10.1] vs. cold-to-warm 11.6 [11.2–12.6] mmol/L, *p* > 0.99). For the HOPE+NMP group, there was a slow concentration increase during the hypothermic phase and then a drop (related to the perfusate fluid replacement). After 2 hours of NMP, the livers started to consume glucose from the perfusate and both groups reached similar figures at the end of 6 hours perfusion (HOPE+NMP 27.1 [14.1–40.0] vs. cold-to-warm 24.5 [21.9–31.8] mmol/L, *p* = 0.15). Bile production at 6 hours of perfusion was similar for both groups (HOPE+NMP 5.2 mL/kg liver vs. cold-to-warm 3.2 mL/kg liver). Fig 3 outlines these results in further detail.

Finally, all five livers submitted to the HOPE+NMP protocol were deemed viable at the end of the NMP phase and four of five in the cold-to-warm group. The non-viable liver (cold-to-warm 3) was from a 55-year-old brain-stem dead donor with a donor risk index of 2.0 and more than 13 hours of cold ischaemia time. Information surrounding the fulfilment of the viability criteria are presented in Table 3.

**Table 3:** Viability criteria achievement by the livers in each group.

Criteria	Cold-to-Warm (*n* = 5)	HOPE+NMP (*n* = 5)
Lactate clearance (≤ 2.5 mmol/L)	4 (80%)	5 (100%)
pH > 7.3 perfusate	4 (80%)	2 (40%)
Glucose metabolism	3 (60%)	4 (80%)
HA flow (> 150 mL/min)	5 (100%)	5 (100%)
PV flow (> 500 mL/min)	5 (100%)	5 (100%)
Homogeneous perfusion/ soft parenchyma	5 (100%)	5 (100%)
Bile production	3 (60%)	3 (60%)
**Viable liver**	**4 (80%)**	**5 (100%)**

**Abbreviations**: HOPE: hypothermic oxygenated perfusion of the liver; NMP: Normothermic machine perfusion of the liver; HA: hepatic artery; PV: portal vein

### Histological assessment

**Fig 5:**
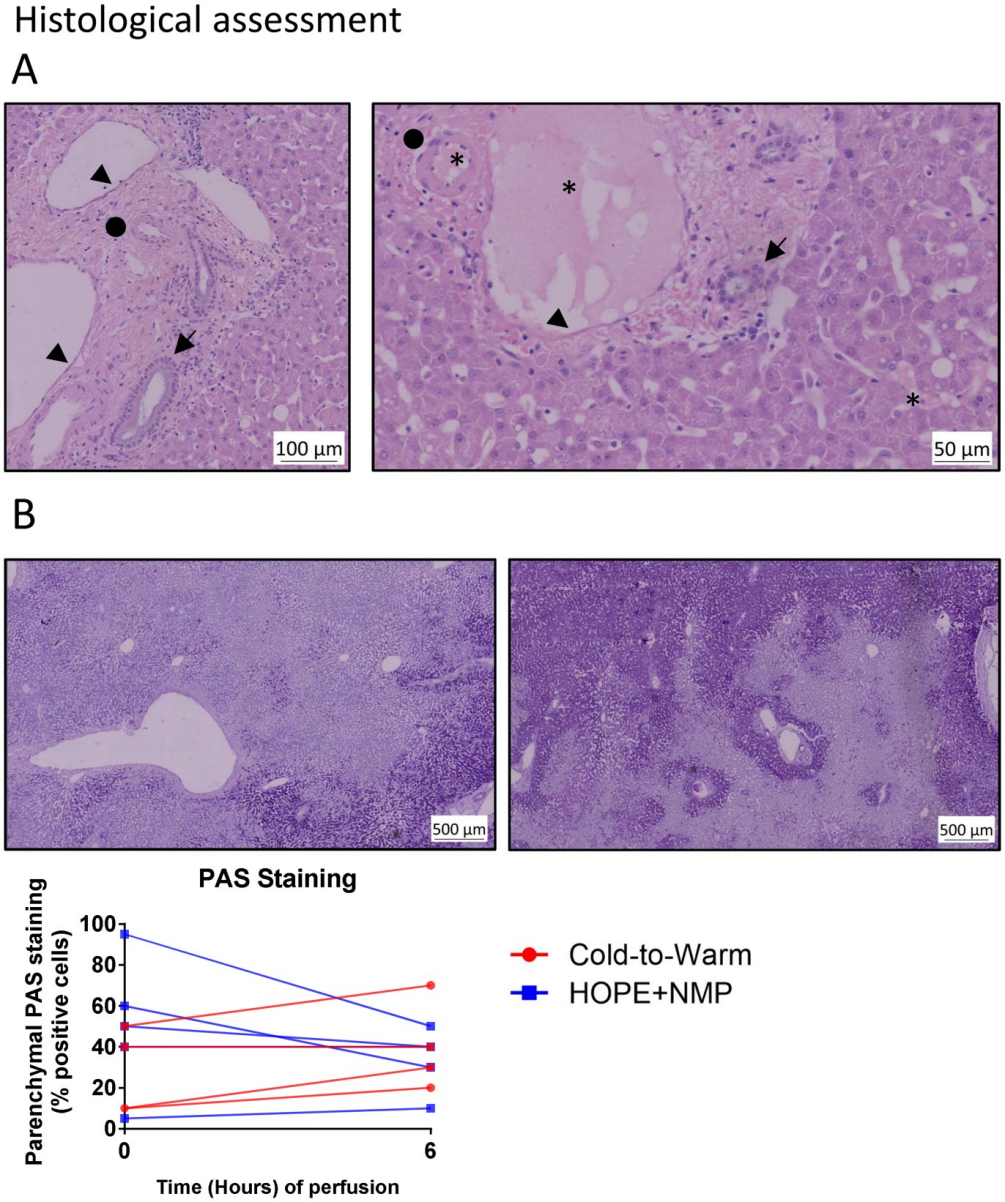
Histological assessment of the perfused organs. Panel A shows two haematoxylin—eosin (H&E)-stained paraffin-embedded sections at the end of the perfusion run. The left-hand picture is a large portal tract showing well-preserved bile duct (arrow), artery (circle) and portal vein (arrowhead). A similar picture was seen for smaller intrahepatic portal tracts in the right-hand picture, where the same well-preserved structures can be identified; and, interestingly, the presence of the Hemopure^®^-based perfusate can be visualized (asterisk) in the vein, artery and even in the sinusoids. Panel B shows the change in glycogen content over time. The first figure on the left shows one liver from the cold-to-warm group at time 0 with severely depleted glycogen stores; at the end of 6 hours of perfusion, this was slightly replenished. The graph compares the dynamic changes in glycogen content between groups.

Histologically, there were no signs of pre-existing acute or chronic liver disease at the beginning of the perfusion in both groups. In terms of severity of macrovesicular steatosis, two livers in the HOPE+NMP were mildly steatotic (5–30%) and two were moderately steatotic (30–60%) in the cold-to-warm group. All other organs were non-steatotic (< 5%). At the end of the 6 hours of perfusion, none of the livers exhibited areas of parenchymal necrosis, congestion or cytoplasmic vacuolization. Additional details are shown in Fig 5.

### Immunohistochemical assessment of oxidative stress and tissue inflammation

**Fig 6:**
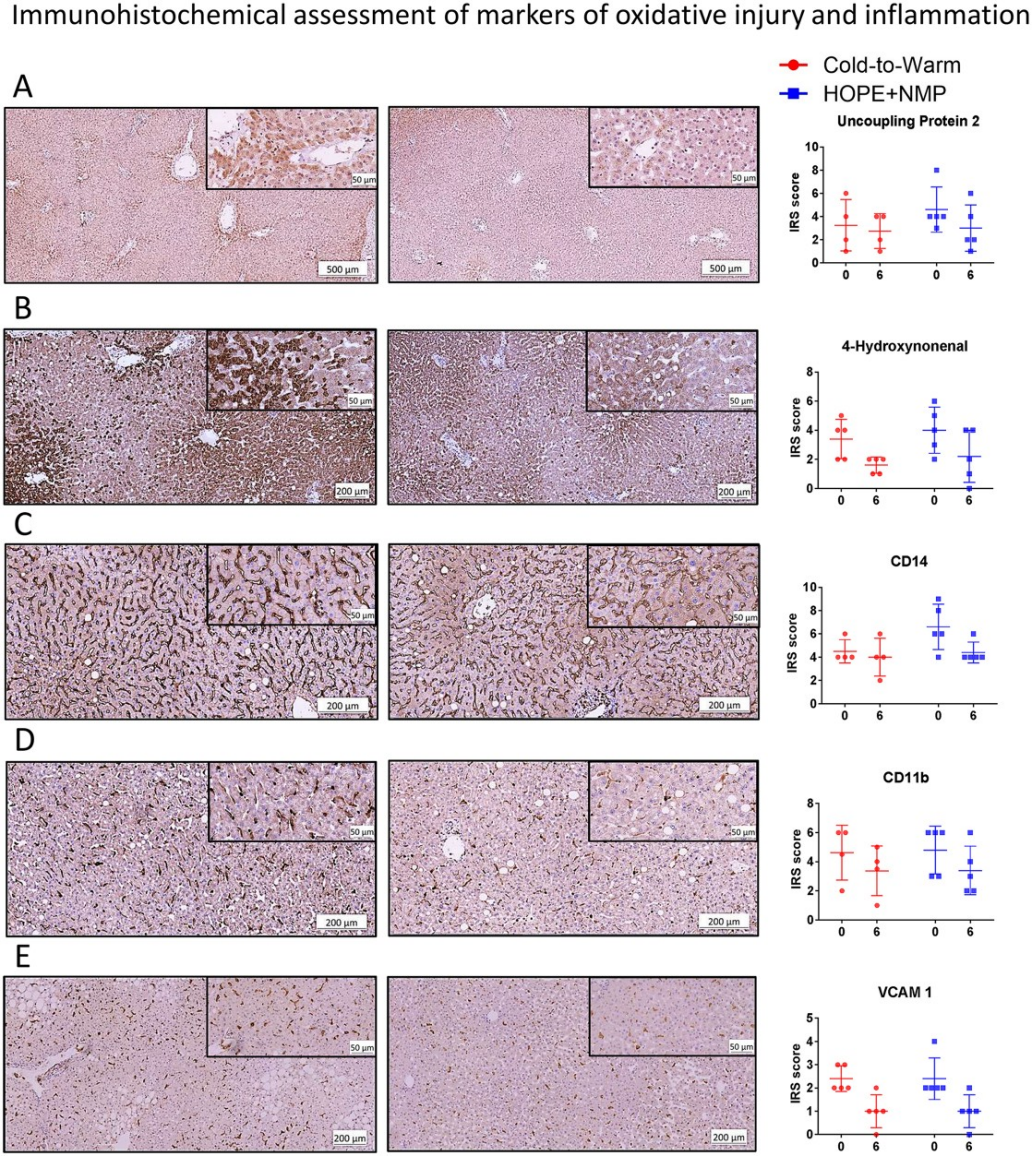
Immunohistochemical assessment of oxidative tissue injury and activation of the inflammatory cascade. Panel A shows the tissue expression of the uncoupling protein 2, a marker for reactive oxygen species (ROS) generation; the left-hand picture shows a moderate diffuse reaction of the staining at time 0, mainly localised at peri-portal areas, which changed to a mild reaction at the end of the 6 hours perfusion. The graph on the right shows the variation in the two groups at these time points. This downregulation in ROS production is associated with a reduction in tissue expression of 4-hydroxynonenal (4-HNE) (Panel B), an established marker for lipid peroxidation during oxidative stress. The lowered oxidative injury decreases the tissue expression of the cluster of differentiation (CD)14 in macrophages (Panel C). CD14 is part of the signalosome of the toll-like receptor 4, which in turn leads to the activation of inflammatory and endothelial cells, perpetuating and aggravating tissue injury. In accordance, a downward trend can be seen during the perfusion in the presence of activated leukocytes, identified by the expression of the CD11b (Panel D) and activated endothelial cells, as represented by the expression of the vascular cell adhesion molecule 1 (VCAM-1) (Panel E). Therefore, both combined protocols exhibited a similar decreasing trend in the expression of markers of ischaemia-reperfusion injury during the perfusion. In the graphs, dots represent individual organs at the time points, and the line is the median of the values for each group.

The expression of tissue markers of oxidative injury and activation of inflammatory cells were comparable for the two groups at the start of the perfusion. During the 6 hours of perfusion, there was an overall decrease in the tissue expression of markers of oxidative injury, UCP2 (*p* = 0.48) and 4-HNE (*p* = 0.43). These markers were present mainly in hepatocytes. Considering downstream activation of the inflammatory cascade, the expression of CD14, part of the signalosome of toll-like receptor 4, decreased in the HOPE+NMP group and was stable for the cold-to-warm group, however, the difference was not statistically significant (*p* = 0.08). Tissue inflammatory response was assessed by the expression of CD11b on activated polymorphonuclear leukocytes (neutrophils and monocytes/macrophages) and VCAM-1 on activated endothelial cells. Its levels of expression decreased similarly between the two groups for both CD11b (*p* = 0.80) and VCAM-1 (*p* = 0.85). Data are presented in Fig 6.

## Discussion

In the present study, we have shown that the combined protocol of HMP and NMP—D-HOPE with sequential slow COR and NMP—using a single HBOC-based perfusate from cold to warm was associated with enhanced ATP synthesis, low tissue expression of markers of oxidative tissue injury and activation of inflammatory cells, similarly to a previously published interrupted combined protocol [8]. Whilst the use of a single perfusate throughout the entire perfusion simplified the logistics of combining HMP and NMP with a slow and controlled rewarming period, it was associated with high vascular resistance and low flow dynamics, mainly during the hypothermic phase. However, this difference did not affect either tissue oxygenation or the enhancement of the bioenergetic status of the organs during the hypothermic phase and allowed effective recovery of the metabolic function of the organs during the normothermic phase.

Recent studies have shown that the combination of a short-term period of HOPE can improve the metabolic parameters of discarded high-risk ECD livers, as evaluated by a subsequent period of NMP [8, 10]. Whilst HMP replenishes cellular energy stores and mitigates ischaemia-reperfusion injury, NMP offers full metabolic support to donor organs, thereby allowing metabolic functioning assessment. Importantly, tests to assess liver function during HMP are still not readily available. The use of nuclear magnetic resonance spectroscopy to determine the bioenergetic status of organs during perfusion or to analyse perfusate composition are possibilities to be further investigated [30–32].

A previous study that performed D-HOPE on human livers using UW-MPS as perfusate reported, at 2 hours of perfusion, median flows of 365 mL/min for PV and 84 mL/min for HA [5]. Our experiments utilised the same perfusion device and applied the same protocol in terms of perfusion pressures and temperature for the D-HOPE phase. However, using an HBOC-based perfusate, we accomplished lower median vascular flows at 2 hours of D-HOPE (150 mL/min and 42 mL/min, respectively). Median vascular arterial resistance at the same time point was proportionally higher for the livers of our experiment (0.4 mmHg/mL/min/kg) than with this previous study (approximately 0.2 mmHg/mL/min/kg). Importantly, similar vascular dynamic parameters can be observed during D-HOPE in the results of the first pilot clinical study using an HBOC-based perfusate for a combination of hypothermic and normothermic MP [9]. Altogether, these findings suggest a possible association between the use of an HBOC-based perfusate at 10 °C, higher vascular resistance and reduced flow. However, during NMP, vascular parameters improved, and they were similar between the groups at the end of the perfusion run.

The association between HBOC and impaired microcirculation has been reported previously and diverse theories were proposed in an attempt to explain this [33]. It has been suggested that, based on their small size, HBOC molecules may be able to permeate into the subendothelial space and acellular haemoglobin consumes nitric oxide (NO) produced by endothelial cells, thereby preventing its biological vasodilatory effect [34, 35]. Alternatively, the low viscosity of HBOCs may affect the regulation of flow within the microcirculation [36, 37]. Mechanistically, the lower shear stress may potentially decrease the production of NO and, therefore, have an impact of vasoconstriction [33, 38]. Finally, the autoregulatory theory proposes that terminal arterioles adjust flow according to needs based on PO_2_ levels [39, 40]. HBOC would potentially be delivering high concentrations of oxygen at this site, paradoxically triggering vasoconstriction. Whether these theories explain the effect observed in our study remains to be seen and requires further investigation. The PO_2_ used for the referred UW-MPS D-HOPE study (60 kPa) [5] was the same as that for our HBOC-based perfusate. However, we do not have comparative data regarding the amount of oxygen delivered at the level of terminal arterioles in each of these models. In addition, in this study, we are unable to comment on the difference in viscosity between Hemopure^®^ and UW-MPS at 10 °C. Till now, the theory that acellular haemoglobin scavenges the NO produced in the subendothelial space remains plausible; however, MetHb levels were stable during HMP, contrary to what would be expected—NO binds to the oxygen sites in haemoglobin, thus generating MetHb.

More importantly, despite these differences in vascular parameters, the enhanced oxygen delivery of HBOCs is likely to have balanced it because adequate oxygen supply was offered to the cells. Both groups showed a reduction in mitochondrial respiration, as represented by a fall in the oxygen requirements associated with lowered CO_2_ production during the hypothermic phase. These findings were associated with enhanced mitochondrial oxidative function and ATP synthesis. This improved mitochondrial oxidative function resulted in lower oxidative tissue injury and activation of inflammatory cells mitigating reperfusion injury during rewarming. Similar findings were reported previously using HOPE and D-HOPE techniques without the use of an oxygen carrier in the perfusate [5, 30]. The initial lactate levels differed slightly between groups at the beginning of the perfusion as a result of varied perfusate composition. Whilst those levels were flat during HOPE, they increased slightly during the first hour of D-HOPE. A similar initial trend was described in previous publications [4, 5, 9, 11]. Importantly, this initial elevation is most likely related to an early flush out of the organs because in isolation, without other markers of reduced perfusion, lactate does not reflect anaerobic metabolism.

Previous clinical studies have reported minor increases in MetHb levels after transfusion of Hemopure^®^ in humans [41, 42]. MetHb is an oxidized form of haemoglobin that cannot bind oxygen and has a reduced ability to release oxygen to tissues [43]. Physiologically, erythrocyte enzymes reduce the MetHb formed, keeping levels lower than 2% [44]. Clinically, levels under 15% are only associated with greyish skin without further complications [43, 44]. In the context of MP, a previous report on a porcine model indicated stable MetHb levels of approximately 2% during subnormothermic MP [13]. We established similarly stable levels during HMP and rewarming, nevertheless, during NMP, MetHb levels rose steadily at a similar rate for both groups, reaching median levels of around 7% at the end of the perfusion. Despite this increase, there was no suggestion of any ischaemic injury to the livers until 6 hours of perfusion. Data on the half-life of the product in this scenario as well as its clearance is still missing. A recent study has proposed that administration of glutathione, vitamin C or addition of extra Hemopure^®^ could potentially be used to overcome issues associated with increasing MetHb concentrations [9], nevertheless, further studies are needed to confirm this hypothesis.

To explore fully the advantages of having a perfusate that could be used in a wide range of temperatures, we incorporate a COR period in the HBOC-based perfusate group. This technique permits avoiding subtle changes in the temperature of the organs up to 20 °C and was shown to be beneficial for mitochondrial function, yielding optimized results in a clinical study [15]. We have employed the same protocol as that clinical study in our experiments, with the major difference being the HBOC-based perfusate instead of Custodiol-N (Dr. Köhler Chemie, Bensheim, Germany) used by the original authors. During this phase, no significant differences were seen in terms of perfusion parameters or liver metabolism features.

The current work has also shown that while an oxygen carrier-free-based perfusate was equally effective during the hypothermic phase, it is possible to use a single HBOC-based perfusate within a range of temperatures in the setting of MP of donor livers. This can be logistically advantageous considering the combination of HMP and NMP techniques because it eliminates the need for perfusate exchange and can increase the clinical applicability of the combined protocol. Despite an effect on flow dynamics with higher vascular resistances and lower flows, the enhanced capacity of oxygen delivery of Hemopure^®^ potentially prevented any harmful effects to the organs. Such findings facilitate and support the safety of a combined protocol of MP using a single HBOC-based perfusate throughout, which can potentially improve the metabolic functions of high-risk discarded ECD livers, and are in accordance with the two clinical series reporting the use of a similar protocol of MP prior to transplantation [9, 11]. In these aforementioned studies, the Groningen group noted the transplantation of ECD donor livers after performing a protocol of D-HOPE+COR+NMP using a single HBOC-based perfusate; the authors concluded that this method is feasible, that HBOC may possibly be a safe alternative to red blood cells for *ex situ* MP of the liver, and also suggested that this protocol may increase the number of transplantable livers [9, 11]. Despite similarities, the present study differentiates itself positively because it brings unique methodological features (strict adherence to originally described D-HOPE and COR protocols, including perfusion pressures [5, 15]), applies alternative clinically validated viability criteria during NMP for transplantation of discarded human donor livers [19], uses as a control group a previously published combined protocol of HMP and NMP [8], and, more importantly, undertakes a careful histopathological analysis of hepatocyte injury and immunohistochemical assessment of oxidative stress and tissue inflammation, as well as assessment of tissue ATP levels.

The present study does have limitations. First, the scarcity of human donor organs for research limits the sample size, which, in turn, limits the power of the statistical analysis. Second, the organs were not transplanted, therefore viability remains theoretical, and the singularity of human donor livers makes the experimental groups imperfectly matched. The uninterrupted cold-to-warm MP protocol using an HBOC-based perfusate was compared with a previously published combination of HMP+NMP, and the differences in the perfusate composition and technique employed limited isolated comparison of HMP techniques exclusively. Replacement of the perfusate, which may reduce toxic metabolites (e.g., cytokines), is a potential confounding factor for the HOPE+NMP group; however, this analysis was beyond the scope of the present study. We were unable to evaluate transaminases or other markers of oxidative stress in the perfusate because the free haemoglobin concentration in the Hemopure^®^-based perfusate exceeded the maximum haemolytic index tolerance for our hospital clinical laboratory assessment, and we were unable to measure those subsequently by alternative methods from rethawed frozen samples. In addition, the absolute values and proportional ATP increase were lower than those reported by other studies performing D-HOPE on human livers [5]. This is most likely related to a longer cold ischaemia time in our study because this can underestimate ATP readings. Moreover, the absence of further data regarding bile duct histology, bile properties at time points other than 6 hours allied to the inability of some of the organs to produce bile, limit our interpretation of suggested markers for bile duct injury during MP, such as bile pH [45, 46].

## Conclusion

Despite the changes in flow dynamics and vascular resistance, the uninterrupted combined protocol of HMP and NMP using an HBOC-based perfusate from cold to warm was associated with enhanced ATP synthesis, low tissue expression of markers of oxidative tissue injury and activation of inflammatory cells, similarly to an interrupted combined protocol. However, the use of a single perfusate facilitated the combination of HMP and NMP and eliminated entirely any concerns of exposing the organs to additional unnecessary ischaemia time required for perfusate exchange and, thus, may potentially increase the clinical applicability of the combined protocol.

## Supporting information

S1 Table Machine perfusion fluid constitution. The perfusion fluid for the normothermic phase of the combined perfusion protocols.
(DOCX)

## Abbreviations

4-HNE: 4-hydroxynonenal
ATP: Adenosine triphosphate
CD11b: Cluster of differentiation 11b
CD14: Cluster of differentiation 14
COR: Controlled oxygenated rewarming
DCD: Donation after circulatory death
D-HOPE: Dual hypothermic oxygenated perfusion
ECD: Extended criteria donors
HA: Hepatic artery
HBOC: Polymerised bovine haemoglobin-based oxygen carrier
HMP: Hypothermic oxygenated machine perfusion
HOPE: Hypothermic oxygenated perfusion
IRS: Immunoreactive score
MP: *Ex situ* machine perfusion of the liver
NMP: Normothermic machine perfusion
PV: Portal vein
ROS: Reactive oxygen species
UCP2: Uncoupling protein 2
UW-MPS: Belzer MPS^®^ UW Machine Perfusion Solution
VCAM-1: Vascular cell adhesion molecule 1
