# Correction: The impact on the bioenergetic status and oxidative-mediated tissue injury of a combined protocol of hypothermic and normothermic machine perfusion using an acellular haemoglobin-based oxygen carrier: The cold-to-warm machine perfusion of the liver

**DOI:** 10.1371/journal.pone.0230062

**Published:** 2020-02-27

**Authors:** Yuri L. Boteon, Richard W. Laing, Andrea Schlegel, Lorraine Wallace, Amanda Smith, Joseph Attard, Ricky H. Bhogal, Gary Reynolds, M. Thamara PR Perera, Paolo Muiesan, Darius F. Mirza, Hynek Mergental, Simon C. Afford

The ninth author's initials are indexed incorrectly in PubMed. The correct initials are: Perera MTPR.
