# Supplementary material for: The impact on the bioenergetic status and oxidative-mediated tissue injury of a combined protocol of hypothermic and normothermic machine perfusion using an acellular haemoglobin-based oxygen carrier: The cold-to-warm machine perfusion of the liver
Source: PLoS One. 2019 Oct 23;14(10):e0224066. doi: 10.1371/journal.pone.0224066 (PMC6808429; doi:10.1371/journal.pone.0224066)
Supplement: S1 Table — The perfusion fluid for the normothermic phase of the combined perfusion protocols. (DOCX) [file pone.0224066.s001.docx]

**The impact on the bioenergetic status and oxidative-mediated tissue injury of a combined protocol of hypothermic and normothermic machine perfusion using an acellular haemoglobin-based oxygen carrier: the cold-to-warm machine perfusion of the liver**

**Authors**

Yuri L. Boteon, Richard W. Laing, Andrea Schlegel, Lorraine Wallace, Amanda Smith, Joseph Attard, Ricky H. Bhogal, Gary Reynolds, M Thamara PR Perera, Paolo Muiesan, Darius F. Mirza, Hynek Mergental, Simon C. Afford

**Supporting Information – Table of Content**

1. **Complementary Methods**
   1. **Technical aspects of the protocols of ex situ machine perfusion of the liver employed in the study**
      1. **Hypothermic oxygenated perfusion (HOPE)**
      2. **Dual hypothermic oxygenated perfusion (D-HOPE)**
      3. **Controlled oxygenated rewarming (COR)**
      4. **Normothermic machine perfusion (NMP)**
   2. **Immunohistochemistry**
   3. **Adenosine trisphosphate quantification**
   4. **Oxygen content in the perfusate equation**
2. **S1 Table**

**Complementary methods**

**Technical aspects of the protocols of ex situ machine perfusion of the liver employed in the study**

**Hypothermic oxygenated perfusion (HOPE)**

The HOPE phase was performed via portal vein (PV) only, using three litres of Belzer MPS^®^ UW Machine Perfusion Solution (UW-MPS) (Bridge to Life, London, UK), with the temperature set at 10 °C. The target flow was 0.1 mL/min/g of liver with a maximum pressure of 3 mmHg. The target oxygen perfusate pressure (pO_2_) was 80–100 kPa. After 2 hours of HOPE, the perfusion was stopped, and the liver temporarily placed on ice. The system was then drained and subsequently refilled with the haemoglobin-based oxygen carrier (HBOC)-based perfusate (Hemopure^®^ - haemoglobin glutamer-250 [bovine]; HBOC-201, Hemoglobin^®^ Oxygen Therapeutics LLC, Cambridge, USA) for the normothermic phase of the combined protocol of perfusion. The hepatic artery (HA) and common bile duct were cannulated and normothermic machine perfusion (NMP) commenced. The perfusion fluid exchange until the start of NMP took on average 20 minutes.

**Dual hypothermic oxygenated perfusion (D-HOPE)**

Before initiation of the perfusion, the cystic duct was ligated, and the common bile duct cannulated with a 12 French biliary drain. The PV was cannulated with the designated cannula provided by the manufacturer and the coeliac trunk with a 16 French plastic tube. The D-HOPE perfusion was performed via PV and HA, using a perfusate consisted of 1000 mL (4 units) Hemopure^®^ complemented with human albumin solution. Temperature was set at 10 °C and the target of flow on the PV was 0.1 mL/min/g of liver with a maximum pressure of 5 mmHg. The pressure on the HA was 20-25 mmHg and oxygen offered to achieve a pO_2_ of 60 kPa. After two hours of D-HOPE, livers were slowly rewarmed using the COR technique.

**Controlled oxygenated rewarming (COR)**

This phase was sequential to the D-HOPE period and lasted for 1 hour. At the beginning, PV pressure was increased to 5 mmHg and HA pressure to 30 mmHg. The temperature was raised 3 °C each 20 minutes during the period targeting 20 °C until the end of 1 hour. At the beginning of this phase the HBOC-based perfusate was supplemented with antibiotics and other complements (specified in S1 Table).

**Normothermic machine perfusion (NMP)**

The perfusion fluid for the NMP phase was Hemopure^®^-based as detailed in S1 Table. The UW-MPS was changed for the NMP perfusate before commencement of the NMP for the HOPE+NMP group. The cold-to-warm group already had this perfusate circulating in the system and, therefore, there was no change. Both groups had, at the start of the NMP phase, a perfusate temperature of 20 °C , pressures of 5 mmHg on the PV and 30 mmHg on the HA. From there, the temperature was raised incrementally to 37 °C . The target of flow was 0.25 mL/min/g liver tissue on the arterial side and 0.75 mL/min/g liver tissue on the venous circuit. To achieve these flows, perfusion pressures on the device were adjusted between 30-50 mmHg (mean pressure) on the arterial side and 8-10 mmHg on the PV. The partial pressure of oxygen in the perfusate was set at 40 kPa in both groups at this phase.

**Immunohistochemistry**

Immunohistochemistry was performed on paraffin-embedded sections using Specie specific ImmPRESS™ (Peroxidase) Excel Amplified HRP Polymer Staining Kit (Vector laboratories, Burlingame, CA, USA). The primary antibodies used were:

- anti-uncoupling protein 2 (UCP2) antibody (ab203244 – Abcam, Cambridge, UK) rabbit polyclonal at 1/600 dilution.
- anti-cluster of differentiation 14 (CD14) antibody (ab36595 – Abcam, Cambridge, UK) mouse monoclonal at 1/500 dilution.
- anti-cluster of differentiation 11b (CD11b) antibody (ab52478 – Abcam, Cambridge, UK) rabbit monoclonal at 1/200 dilution.
- anti-4-hydroxynonenal (4-HNE) antibody (ab46545; Abcam, Cambridge, MA, USA) rabbit polyclonal IgG at 1:200 dilution.
- anti-vascular cell adhesion molecule 1 (VCAM-1) antibody (SAB1406579 - Sigma-Aldrich Inc, St Louis, MO, USA) mouse polyclonal at 1/400 dilution.

All sections were wholly digitalized using the Axio Scan.Z1. Four areas at a magnification of 400x were randomly selected using the ZEN image analysis software. Areas of tissue edge were not included due to artefacts that could confound the analysis. Thereafter, the four random images were analysed for overall expression of the staining.

**Adenosine trisphosphate** **quantification**

Adenosine trisphosphate (ATP) concentration was determined using the ATP Bioluminescent Kit (FL-AA - Sigma-Aldrich Inc, St Louis, MO, USA). One-hundred milligrams of frozen liver tissue was immediately homogenised in 1 mL SONOP Buffer (0.372 g EDTA in 130 mL ddH_2_O [adjusted to pH 10.9 with NaOH] = 370 mL of 96% Ethanol) using the gentleMACS^TM^ Dissociator. Particulates were removed by centrifugation at 13,000xg. The protein concentration was determined in the supernatant with the use of a Pierce^TM^ BCA^TM^ Protein Assay kit (Thermo Fisher Scientific, Rockford, Illinois, USA) and the concentration adjusted to 300 µg/mL protein with the SONOP buffer. Samples were then diluted 10-fold in 100 µM phosphate buffer and then the ATP concentration determined. Concentrations were determined from a calibration curve on the same plate, corrected for amount of protein and expressed as nmol/g protein.

**Oxygen content in the perfusate equation**

O_2_ content =  (pO_2_ × K)+(sO_2_ × Hb × c), pO_2_ in kPa, K equals 0.027 for O_2_ in water at 37 °C, Hb in mmol/L and c equals 91.12 mLO_2_/mmol for the oxygen binding capacity of haemoglobin.

**S1 Table: Machine perfusion fluid constitution.** The perfusion fluid for the normothermic phase of the combined perfusion protocols.

| 1000 mL (4 units) acellular oxygen haemoglobin carrier Hemopure^®^  (hemoglobin glutamer-250-bovine; HBOC-201, Hemoglobin Oxygen Therapeutics LLC, Cambridge, MA) |
| --- |
| 1000 mL 5% w/v human albumin solution (Alburex 5, CSL Behring GmbH, Germany) |
| 10,000 IU heparin (Wockhardt, UK) |
| 30 mL sodium bicarbonate 8.4% (B. Braun Medical Limited, UK) |
| 10 mL calcium gluconate 10% |
| 500 mg vancomycin (Wockhardt, UK) |
| 60 mg gentamicin (Cidomycin, Sanofi, UK) |
| 50 mL 10% v/v Aminoplasmal (B.Braun Medical Limited, UK) |
| 0.2 mL Cernevit (Baxter Healthcare Ltd., UK) |
| 0.1 mg phytomenadione (Konakion, Roche Products Ltd, UK) |
| Epoprostenol (Flolan, GlaxoSmithKline, UK, 2 µg/mL) continuous infusion commencing at 4 mL/hour |

Abbreviations: MA- Massachusetts; UK- United Kingdom.
